# CD4 Donor Lymphocyte Infusion Can Cause Conversion of Chimerism Without GVHD by Inducing Immune Responses Targeting Minor Histocompatibility Antigens in HLA Class II

**DOI:** 10.3389/fimmu.2018.03016

**Published:** 2018-12-18

**Authors:** Peter van Balen, Cornelis A. M. van Bergen, Simone A. P. van Luxemburg-Heijs, Wendy de Klerk, Esther H. M. van Egmond, Sabrina A. J. Veld, Constantijn J. M. Halkes, Jaap-Jan Zwaginga, Marieke Griffioen, Inge Jedema, J. H. Frederik Falkenburg

**Affiliations:** ^1^Department of Hematology, Leiden University Medical Center, Leiden, Netherlands; ^2^Center for Clinical Transfusion Research, Sanquin Research, Leiden, Netherlands; ^3^Department of Immunohematology and Blood Transfusion, Leiden University Medical Center, Leiden, Netherlands

**Keywords:** HLA class II, CD4 donor lymphocyte infusion, minor histocompatibility antigen, allogeneic stem cell transplantation, graft-vs.-tumor reactivity

## Abstract

Under non-inflammatory conditions HLA class II is predominantly expressed on hematopoietic cells. Therefore, donor CD4 T-cells after allogeneic stem cell transplantation (alloSCT) may mediate graft-vs.-leukemia reactivity without graft-vs.-host disease (GVHD). We analyzed immune responses in four patients converting from mixed to full donor chimerism without developing GVHD upon purified CD4 donor lymphocyte infusion (DLI) from their HLA-identical sibling donor after T-cell depleted alloSCT. *In vivo* activated T-cells were clonally isolated after CD4 DLI. Of the alloreactive T-cell clones, 96% were CD4 positive, illustrating the dominant role of CD4 T-cells in the immune responses. We identified 9 minor histocompatibility antigens (MiHA) as targets for alloreactivity, of which 8 were novel HLA class II restricted MiHA. In all patients, MiHA specific CD4 T-cells were found that were capable to lyse hematopoietic cells and to recognize normal and malignant cells. No GVHD was induced in these patients. Skin fibroblasts forced to express HLA class II, were recognized by only two MiHA specific CD4 T-cell clones. Of the 7 clones that failed to recognize fibroblasts, two targeted MiHA were encoded by genes not expressed in fibroblasts, presentation of one MiHA was dependent on HLA-DO, which is absent in fibroblasts, and T-cells recognizing the remaining 4 MiHA had an avidity that was apparently too low to recognize fibroblasts, despite clear recognition of hematopoietic cells. In conclusion, purified CD4 DLI from HLA-identical sibling donors can induce conversion from mixed to full donor chimerism with graft-vs.-malignancy reactivity, but without GVHD, by targeting HLA class II restricted MiHA.

## Introduction

Allogeneic stem cell transplantation (alloSCT) provides a potentially curative therapy for patients with a variety of hematologic malignancies. However, acute graft-vs.-host disease (GVHD) and treatment of GVHD remain major causes of transplant related morbidity and mortality. The most efficient method to prevent GVHD is T-cell depletion (TCD) of the graft. AlloSCT regimens using infusion of positively selected CD34 cells or using the CD52 antibody Alemtuzumab for TCD have demonstrated efficient engraftment and reduced acute GVHD (1–4). However, TCD substantially impairs post-transplant anti-viral and anti-tumor immunity (4–6). Due to the reduced antitumor effect of TCD grafts, pre-emptive post-transplant donor lymphocyte infusion (DLI) may be needed for treatment of mixed chimerism or persistent disease. Indeed, DLI after alloSCT can mediate the beneficial graft-vs.-leukemia (GVL) reactivity, but frequently still at the cost of GVHD (7–10).

Separation of GVL from GVHD may be achieved by infusion of alloreactive donor T-cells that recognize patient hematopoietic cells, including the malignant cells, but not other tissue cells of the patient or donor hematopoietic cells (11). Following HLA matched alloSCT, infusion of donor CD8 T-cells recognizing minor histocompatibility antigens (MiHA) selectively expressed in hematopoietic cells may result in destruction of patient hematopoietic cells including the malignant cells, without harming normal tissues (12–15). MiHA are polymorphic peptides derived from genes containing single nucleotide polymorphisms which differ between donor and recipient and can be recognized in the context of (self) HLA. Although infusions of patient hematopoiesis directed donor CD8 T-cells are being explored, the numbers of known hematopoiesis restricted MiHA that can be targeted are too limited for broad application (16–19).

In contrast to HLA class I, constitutive expression of HLA class II molecules is predominantly restricted to normal and malignant hematopoietic cells (20–23). Therefore, infusion of donor CD4 T-cells recognizing HLA class II restricted MiHA may result in selective recognition of recipient normal and malignant hematopoietic cells, thereby inducing GVL without GVHD even if MiHA are targeted that are encoded by genes that are broadly expressed in recipient tissues (24–28). This is supported by previous findings that, following an HLA 10/10 matched, but HLA-DPB1 mismatched TCD alloSCT, allo-HLA-DP directed CD4 T-cells can cause GVL without GVHD (29, 30). However, under inflammatory circumstances, expression of HLA class II is significantly upregulated on non-hematopoietic cells, making these tissues susceptible to recognition by CD4 T-cells. Indeed, during viral infections, allo-HLA-DP directed CD4 T-cells can induce GVHD (31), suggesting that also CD4 T-cells directed against broadly expressed HLA class II restricted MiHA may cause GVHD when HLA class II expression is upregulated on non-hematopoietic cells. However, we recently demonstrated that not all MiHA encoded by broadly expressed genes are adequately presented in HLA class II on non-hematopoietic cells due to absence of HLA-DO, the natural inhibitor of HLA-DM (32). Furthermore, we demonstrated that selective GVL also depends on the magnitude and diversity of alloreactive T-cell responses and not only on tissue distribution of the MiHA that are targeted (33). Thus, even under inflammatory conditions, GVHD target tissues may not always be damaged by CD4 T-cells recognizing MiHA encoded by broadly expressed genes.

Within an ongoing clinical trial, initiated in the Leiden University Medical Center, treating patients 3 months after TCD alloSCT with an HLA-identical sibling donor with purified donor CD4 T-cells with primary aim to improve immune reconstitution (34), we observed hematopoiesis restricted immune responses, as illustrated by conversion from mixed to full donor chimerism, without GVHD in four patients. We identified alloreactive CD4 T-cells recognizing HLA class II restricted MiHA in all four patients without the presence of alloreactive CD8 T-cells in 3 of 4 patients. Using whole genome association scanning, 9 HLA class II MiHA were identified as targets for recognition. No GVHD was induced by these MiHA specific CD4 T-cells, corresponding to lack of HLA class II expression on GVHD target tissues under steady state conditions. Even after upregulation of HLA class II on non-hematopoietic cells, recognition was relatively restricted to hematopoietic cells, although the majority of the identified MiHA were encoded by genes that are broadly expressed. MiHA specific CD4 T-cells from all four patients were able to recognize malignant cells. Therefore, CD4 DLI after TCD alloSCT may be an attractive strategy to separate GVL from GVHD in patients transplanted with an HLA-identical sibling donor.

## Material and Methods

### Patients

At the Leiden University Medical Center (LUMC) patients to be transplanted with mobilized peripheral blood stem cells from HLA-identical sibling donors are treated with a conditioning regimen consisting of fludarabine (50 mg/m^2^ orally from day −10 to −5), busulfan (0.8 mg/kg iv four times a day on day −7 and −6) and alemtuzumab (15 mg iv on day −4 and −3) in case of a non-myeloablative conditioning, or consisting of cyclophosphamide (60 mg/kg iv on day −6 and −5) and total body irradiation (9 Gy on day −1) in case of a myeloablative conditioning regimen. Stem cell grafts are T-cell depleted by addition of 20 mg alemtuzumab to the bag before administration and no post-transplant immune suppression is applied (4, 35). Three months after TCD alloSCT, in the absence of overall grade II or more GVHD according to the Glucksberg-Seattle classification, (36) patients are eligible for treatment with infusion of purified donor CD4 T-cells at a dose of 10^6^ cells per kilogram body weight in the setting of a randomized clinical trial (EudraCT Number: 2008-001447-19). This ongoing phase II open-label single-center randomized clinical trial was approved by the LUMC Institutional Review Board and national authorities. Purified CD4 DLI cell products were manufactured by positive selection using CD4 Reagents (Miltenyi Biotec, Bergisch Gladbach, Germany) and CliniMACS System (Miltenyi Biotec) according to the manufacturer's instructions. The study aims to evaluate the immunological effects of prophylactic DLI of purified CD4 T-cells early after TCD alloSCT. The primary objective is to evaluate whether CD4 DLI improves immunological recovery within 6 months after alloSCT. Secondary objectives are the evaluations of influence on chimerism and disease status after CD4 DLI. Since the trial is still ongoing, we do not describe the results of the entire clinical trial in this manuscript, but only the results of an in depth analysis of immune responses occurring in a selection of trial participants.

### Isolation, Expansion, and Selection of T-Cell Clones

Peripheral blood, bone marrow, and skin biopsies for the generation of fibroblasts were obtained from the patients, their donors, and third party healthy individuals after approval by the LUMC Institutional Review Board and informed consent according to the Declaration of Helsinki. Mononuclear cells were isolated using Ficoll separation and cryopreserved.

To isolate *in vivo* activated T-cells, peripheral blood mononuclear cells (PBMC) obtained after CD4 DLI or 6 weeks after randomization in case patients did not receive CD4 DLI, were stained with antibodies against CD8 (Alexa Fluor, Invitrogen/Caltag, Buckingham, UK), CD4 (FITC, BD/Pharmingen, Breda, Netherlands), CD14 (APC, ITK/Biolegend, Uithoorn, Netherlands), and HLA-DR (PE, BD). HLA-DR^+^ CD8 and HLA-DR^+^ CD4 T-cells were sorted single cell into 96-well U-bottomed plates (Corning, Amsterdam, Netherlands) or 384-well flat bottomed plates (Greiner Bio-One, Alphen a/d Rijn, Netherlands). T-cell clones were expanded using Iscove's modified Dulbecco's medium (IMDM, Lonza BioWhittaker, Verviers, Belgium) with 5% pooled human serum, 5% fetal bovine serum (FBS, Gibco Invitrogen, Bleiswijk, Netherlands), 100 IU/ml Interleukin 2 (Chiron, Amsterdam, Netherlands), 2 ng/ml Interleukin 7 (Miltenyi Biotec), 2 ng/ml Interleukin 15 (Miltenyi Biotec), 0.8 μg/ml phytohemagglutinin (Murex Biotec Limited, Dartford, UK) and 25–50 × 10^3^ irradiated third party PBMC as feeder cells. Proliferating T-cell clones were restimulated every 10–14 days and tested for reactivity against patient and donor derived EBV-LCL. After overnight incubation of 2 × 10^4^ patient or donor derived EBV-LCL with 2 × 10^3^ T-cells, recognition was measured by IFNγ ELISA according to the manufacturer's instructions (Sanquin Reagents, Amsterdam, Netherlands). A T-cell clone was determined to be alloreactive when at least 500 pg/ml IFNγ was produced after incubation with patient derived EBV-LCL and no IFNγ was produced after incubation with donor derived EBV-LCL.

### HLA Restriction and TCRBV Usage of Alloreactive T-Cells

To determine whether HLA-DR, HLA-DQ, or HLA-DP was the HLA restriction molecule for recognition by alloreactive CD4 T-cells, patient derived EBV-LCL were pre-incubated with saturating concentrations of monoclonal antibodies (MoAb) against HLA class II (PdV5.2), HLA-DR (B8.11.2), HLA-DQ (SPVL3), or HLA-DP (B7.21) for 30 min at room temperature before addition of the T-cells, and inhibition of IFNγ production was determined. T-cell receptor-β variable chain (TCRBV) usage of the T-cell clones was investigated by flow cytometry using specific monoclonal antibodies as provided with the TCRBV repertoire kit (Beckman Coulter).

### MiHA Identification by Whole Genome Association Scanning

The method of whole genome association scanning (WGAS) using an HLA transduced panel of third party EBV-LCL was described earlier (37). In short, 48–116 third-party EBV-LCL were transduced with one of the possible HLA restriction molecules. The transduced EBV-LCL were incubated with the alloreactive CD4 T-cells and IFNγ production was measured using ELISA. The presence or absence of recognition of the different EBV-LCL was compared with the EBV-LCL genotype data of over one million single nucleotide polymorphisms (SNPs) in order to find an association between the recognition and the presence of a certain SNP. If association with a missense SNP was found, patient and donor variant peptides encoded by the SNP region were synthesized. If incubation of donor derived EBV-LCL loaded with patient variant peptide, titrated in a concentration from 10^−4^ to 10^−10^ M, resulted in IFNγ production by the T-cell clone, this peptide was confirmed to be the MiHA.

### Cytotoxicity of MiHA Specific CD4 T-Cells

Cytotoxic capacities of alloreactive CD4 T-cells was analyzed following incubation of 2.5 × 10^5^ T-cells with 2.5 × 10^4^ EBV-LCL target cells labeled with PKH26 Red Fluorescent (Sigma-Aldrich, Zwijndrecht, Netherlands). Target cell survival after 24 h was measured by flowcytometric cell counting of the target cells using Flow-Count fluorospheres (Beckman Coulter, Woerden, Netherlands) (38). Percentage cell lysis was calculated by the formula 100–100^*^(total number of surviving target cells after incubation with T-cells/total number of target cells without incubation with T-cells). Statistical analysis was performed using Mann-Whitney *U*-test and significance was defined by *p* < 0.05.

### Recognition of PHA Blasts, Malignant Cells and Skin Derived Fibroblasts

To investigate the recognition of patient derived activated T-cells, T-cells isolated from the patient before alloSCT were treated with phytohemagglutinin (PHA blasts) and incubated with MiHA specific CD4 T-cells in a stimulator to responder ratio of 5:1. To investigate the recognition of malignant cells, MiHA specific CD4 T-cells were incubated with primary malignant cells preferably of the patient. If not available, third party cells representative for the malignancy of the patient were used, like bone marrow cells of a patient suffering from chronic myeloid leukemia and multiple myeloma cell lines UM-6 (CVCL-W395), UM-3 (CVCL-W394), RPMI8226 (ATCC CRM-CCL-155), or U266 (ATCC-TIB-196). The malignant cells expressed the MiHA and HLA restriction molecule of interest endogenously or after retroviral transduction. To investigate recognition of skin derived fibroblasts, 4 × 10^3^ fibroblasts were incubated with 2 × 10^3^ MiHA specific CD4 T-cells. Fibroblasts were induced to express HLA class II molecules on the cell surface by culturing them with 200 IU/ml IFNγ (Boehringer Ingelheim, Rijnland-Palts, Germany) for 5 days in Dulbecco's Modified Eagle Medium (DMEM, Lonza BioWhittaker) supplemented with 10% FBS. Because peptide processing in HLA class II could be different in fibroblasts compared to EBV-LCL due to the absence of HLA-DO in fibroblasts, the role of HLA-DO in the recognition of fibroblasts by alloreactive CD4 T-cells was studied using fibroblasts that were retrovirally transduced with HLA-DOα and HLA-DOβ as described previously (32). HLA class II expression on target cells was analyzed using flowcytometry with monoclonal antibodies against HLA-DR (PE, IgG2a, L-243, BD), HLA-DQ (PE, IgG2a, 1a3, Bio-Connect, Huissen, The Netherlands) and HLA-DP (PE, IgG3, B7.21, Bio-Connect) and T-cell recognition was analyzed by measurement of IFNγ production.

### Gene Expression Profiles

To investigate whether the MiHA encoding genes are expressed in hematopoietic cells only or also in non-hematopoietic cells, quantitative RT-PCR was performed using TaqMan Assays (ThermoFisher, Breda, Netherlands). Expression of the gene of interest was corrected for expression of household reference genes GAPDH and B-actin.

## Results

### Patient Characteristics

The first 15 patients who were treated with CD4 DLI in the intervention arm of the clinical trial, did not develop GVHD, except for patient D, who developed mild skin GVHD (overall grade I) 3 months after infusion, which resolved completely after start with topical steroids. To investigate whether CD4 DLI could induce an allo-immune response following the infusion in the absence of GVHD, peripheral blood subset chimerism was analyzed. In 11 of 15 patient no conversion of chimerism occurred. However, 4 of 15 patients converted from markedly mixed to full donor chimerism within 3 months after infusion of donor CD4 T-cells, indicating the development of an allo-immune response in a selection of patients after CD4 DLI (Table 1). These four patients were selected for further analysis. Also four patients from the control group were selected. In the control group, no conversion of chimerism was observed, although in patient G improvement of chimerism occurred (Table 1).

**Table 1:** Patients characteristics and chimerism data on different time points from CD4 DLI (patient A–D) or from randomization to the control arm (patients E–H).

**Patient**	**Age, gender**	**Disease**	**Condi-tioning regimen**	**Days from CD4 DLI or randomization**	**Absolute leucocyte count (× 10^**9**^/L)**	**Absolute CD3 count (× 10^**6**^/L)**	**Chimerism (% cells from recipient origin)**			
							**CD8 T-cells**	**CD4 T-cells**	**B cells**	**Granulocytes**
A	46, female	Plasma cell myeloma	NMA	−45 −3 46 95	3.6 5.8 4.2 5.4	529 274 211 420	57 77 60 < 1	9 28 16 < 1	1 4 < 1 < 1	< 1 4 < 1 < 1
B	57, male	Plasma cell myeloma	NMA	−56 −7 35 91	3.5 5.6 4.6 6.2	128 359 529 574	74 2 1 < 1	4 < 1 < 1 < 1	< 1 < 1 < 1	< 1 < 1 < 1 < 1
C	48, male	Chronic myeloid leukemia (blastic phase)	MA	−48 0 50 92	8.6 3.8 3.2 2.7	88 389 254 258	26 90 55 < 1	5 13 5 < 1	< 1 < 1 < 1 < 1	2 5 8 < 1
D	62, male	Acute myeloid leukemia	NMA	−54 −5 45 94	3.2 3.7 4.4 7.7	73 100 196 275	90 86 17 < 1	15 53 8 1	< 1 < 1 < 1 < 1	< 1 < 1 < 1 < 1
E	62, male	Plasma cell myeloma	NMA	0 28 91	1.5 3.6 4.2	326 652 571	60 48 88	7 6 60	< 1 < 1 < 1	< 1 < 1 < 1
F	55, male	Plasma cell myeloma	NMA	0 47 90	4.1 4.8 5.0	91 128 222	55 38 37	20 22 16	< 1 < 1 < 1	< 1 < 1 < 1
G	55, male	Acute myeloid leukemia	NMA	0 49 98	4.6 10.6 5.3	85 67 62	4 < 1 2	27 2 2	1 2 2	1 < 1 1
H	51, female	Plasma cell myeloma	NMA	0 43 97	2.9 3.0 5.8	976 695 454	< 1 < 1 2	1 < 1 1	n.a.	1 < 1 1

*NMA, non-myeloablative; MA, myeloablative; N.a., not available*.

### Isolation of Alloreactive T-Cells

**Figure 1:**
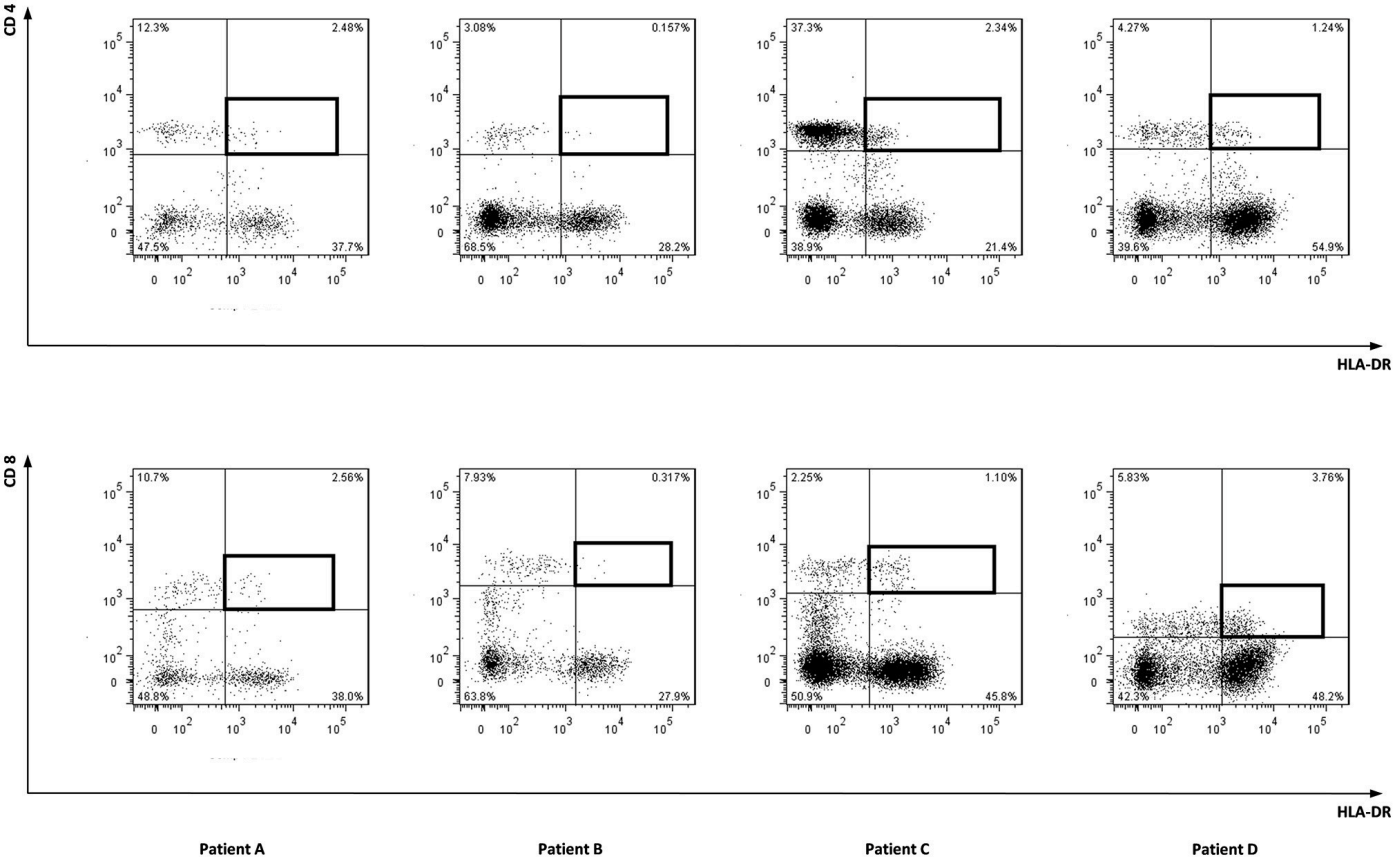
Sorted cell populations. Marked cell populations, being *in vivo* activated HLA-DR positive CD4 or CD8 T-cells from four patients were single cell sorted using flowcytometry using HLA-DR PE, CD4 FITC, and CD8 AF700 monoclonal antibodies.

**Figure 2:**
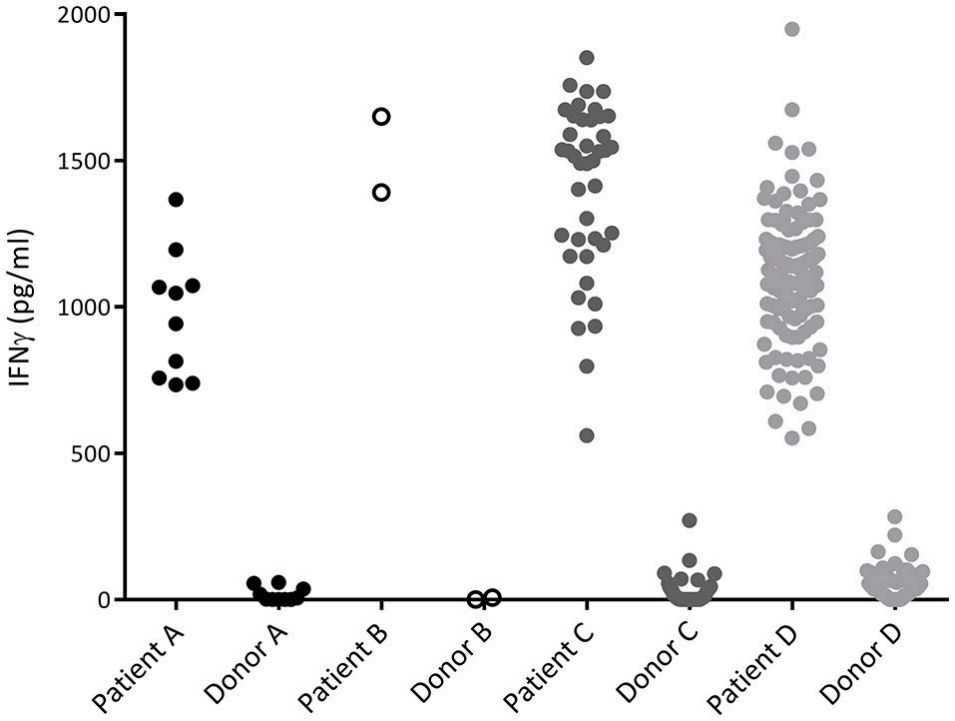
IFNγ production of alloreactive CD4 T-cell clones. Recognition of patient and donor derived EBV-LCL by CD4 T-cells measured by INFγ production after overnight incubation. Each dot represents the INFγ release by one alloreactive CD4 T-cell clone after overnight incubation in a responder to stimulator ratio of 1:10. All CD4 T-cell clones did recognize patient derived EBV-LCL and did not recognize donor derived EBV-LCL and are therefore defined as alloreactive.

To characterize the allo-immune response observed after infusion of donor CD4 T-cells in these four patients, peripheral blood samples were taken during conversion of chimerism. *In vivo* activated, HLA-DR expressing CD4 and CD8 T-cells were clonally isolated using flowcytometric cell sorting (Figure 1) and 5–25% of the single T-cells expanded, resulting in 295–1,396 growing T-cell clones per patient (Table 2). These T-cell clones were tested for allo-reactivity, defined as IFNγ production of at least 500 pg/ml after overnight incubation with patient derived EBV-LCL and absence of IFNγ production after incubation with donor derived EBV-LCL (Figure 2). Reactivity to both patient and donor derived EBV-LCL was interpreted as EBV specific T-cells present in the *in vivo* activated T-cell compartment (Table 2). Total frequencies of 1–28% of the expanded CD4 T-cells were alloreactive as compared to only 0–3% of the expanded CD8 T-cells (Table 2). As controls, four patients randomized to the control arm of the study, who did therefor not receive CD4 DLI and in which conversion of chimerism did not occur, were analyzed 6 weeks after randomization. In these patients 1–5% of expanded CD4 T-cell clones and 3–28% of expanded CD8 T-cell clones were EBV specific. However, only 0–0.3% of expanded CD4 T-cell clones and 0–1% of expanded CD8 T-cell clones were alloreactive (Table 2). These data illustrate that conversion from mixed to full donor chimerism after CD4 DLI was associated with development of dominant alloreactive CD4 T-cell responses.

**Table 2:** Numbers of expanded and alloreactive CD4 and CD8 T-cells after isolation of *in vivo* activated T-cells 6 weeks after CD4 DLI (patient A–D) or randomization (patient E–H).

	**Patient A**	**Patient B**	**Patient C**	**Patient D**		**Patient E**	**Patient F**	**Patient G**	**Patient H**
				**Day 45 after CD4 DLI**	**Day 94 after CD4 DLI**			
Total number of expanded CD4 T-cell clones	332	205	341	384	452	516	360	384	384
Alloreactive CD4 T-cell clones	10	2	41	106	4	1	0	1	0
EBV-LCL reactive CD4 T-cell clones	15	25	1	3	0	20	18	9	4
Total number of expanded CD8 T-cell clones	99	90	66	416	144	538	250	173	344
Alloreactive CD8 T-cell clones	1	0	0	1	4	0	0	2	0
EBV-LCL reactive CD8 T-cell clones	59	33	0	0	1	24	69	5	43

### Identification of HLA Class II Restricted MiHA Using WGAS

**Figure 3:**
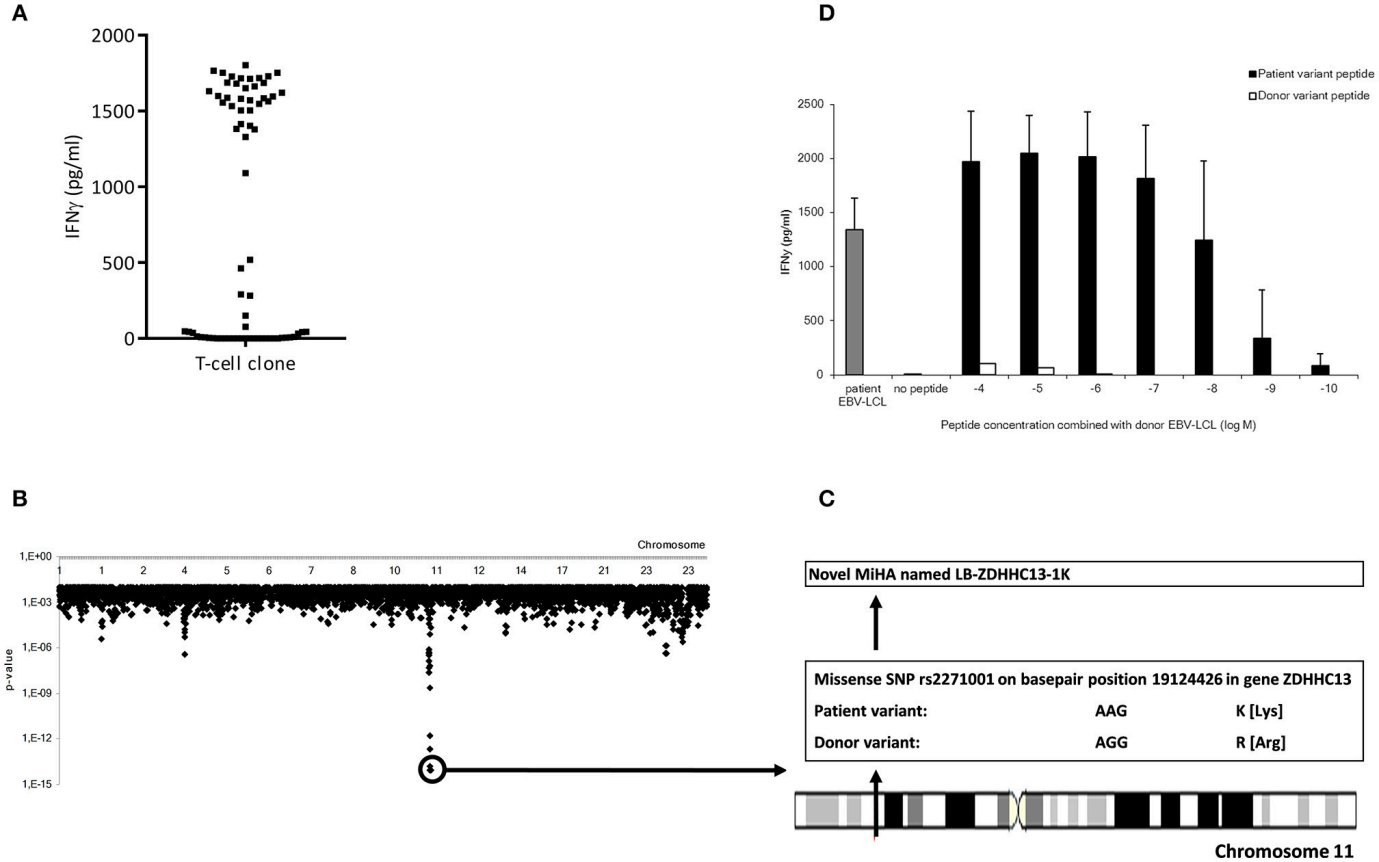
Representative example of the identification of a MiHA by WGAS. **(A)** IFNγ production of clone P07-066 from patient C upon incubation with a panel of 71 SNP genotyped EBV-LCL which were transduced with HLA-DRB1*15:01. **(B)** Identification of associating SNPs on chromosome 11 that are present in EBV-LCL that were recognized by clone P07-066 and absent in EBV-LCL that were not recognized. **(C)** Identification of missense SNP rs2271001 encoding the possible MiHA recognized by clone P07-066. **(D)** IFNγ production by clone P07-066 after incubation with donor EBV-LCL loaded with donor or patient derived allelic peptide variants at titrated concentrations. The patient, but not donor, peptide was recognized by the T-cells, thereby validating the peptide as MiHA.

To characterize the MiHA that are recognized by the alloreactive CD4 T-cell clones, WGAS was applied. We first determined the HLA class II restriction molecules of the T-cell clones using monoclonal antibodies against HLA-DR, DQ, or DP. In addition, TCRBV usage of each T-cell clone was analyzed using flowcytometry. If T-cell clones recognized a target antigen in the context of the same HLA molecule and expressed the same TCRBV, it was considered likely that these clones recognized the same MiHA, and therefore initially one clone was selected for WGAS (Table 3). A representative example of WGAS with a T-cell clone from patient C is shown in Figure 3. When association was found between T-cell recognition and one or more SNPs in the genotypes of a panel of EBV-LCL, patient and donor allelic variants of the peptides encoded by the polymorphic region were synthesized, loaded on donor EBV-LCL and tested for recognition by the T-cell clone. When the MiHA was identified by WGAS and confirmed by T-cell recognition of peptide-pulsed donor EBV-LCL, all other T-cell clones from the same patient were tested for recognition of the identified MiHA. From the T-cell clones that failed to recognize the identified MiHA, one T-cell clone was selected for additional WGAS analysis attempting to further identify additional MiHA. A total number of 16 WGAS experiments were performed, resulting in the identification of 9 MiHA. Eight out of these nine antigens were novel MiHA. In addition to alloreactive CD4 T-cells, 5 alloreactive CD8 T-cell clones were isolated from PBMC of patient D (Table 2). The 4 CD8 T-cell clones isolated 94 days after CD4 DLI all used the same TCRBV, which was different from the TCRBV usage of the CD8 T-cell clone isolated 45 days after CD4 DLI. WGAS resulted in identification of the novel HLA-A^*^03:01 restricted MiHA LB-NADK-1K, which was the target for all 5 isolated CD8 T-cell clones. Table 4 gives an overview of the identified MiHA and their HLA restriction molecules. The HLA-DRB1^*^15:01 restricted MiHA from gene SLC19A1 was earlier described (26), but we found that this MiHA could also be presented and recognized in HLA-DRB1^*^11:01. For 4 T-cell clones from patient A and for 3 T-cell clones from patient D, as well as for 1 T-cell clone from control patient E and 3 T-cell clones from control patient G, MiHA could not be identified due to lack of associating SNPs in WGAS. For all other T-cell clones, however, MiHA were succesfully identified.

**Table 3:** Classification of alloreactive T-cell clones according to HLA restriction molecule and TCR Vβ usage.

**Patient**	**HLA class II restriction molecule^**#**^**	**TCR Vβ usage**	**Identified MiHA**	**Number of clones**
Patient A	undetermined	unknown	–	1^*^
	DQ	2	LB-LILRB1-1I	6^*^
	DQ	2	–	2^*^
	DQ	9	–	1^*^
Patient B	DR	8	LB-ABCA5-1R	1^*^
	DR	13.2	LB-RPS4Y	1^*^
Patient C	DR	2	SCL19A1	21^*^
	DR	5.3	SCL19A1	1^‡^
	DR	unknown	LB-ZDHHC13-1K	10^*^
	DR	unknown	LB-KHNYN-1K	3^*^
	DR	7.1	LB-KHNYN-1K	1^‡^
	DR	9	LB-CTSB-1G	1^*^
	DR	13.2	LB-CTSB-1G	1^‡^
	DR	9	LB-LGALS8-1C	2^*^
	DR	unknown	LB-LGALS8-1C	1^*^
Patient D	undetermined	3	–	2^*^
	DQ	5.1	–	1
	DP	2	LB-LY75-2R	38^*^
	DP	3	LB-LY75-2R	1^‡^
	DP	4	LB-LY75-2R	32^‡^
	DP	8	LB-LY75-2R	6^‡^
	DP	13.2	LB-LY75-2R	3^‡^
	DP	unknown	LB-LY75-2R	27^*^

# *HLA class II restriction molecule was determined using monoclonal antibodies against pan HLA class II (PdV5.2), HLA-DR (B8.11.2), HLA-DQ (SPVL3) and HLA-DP (B7.21)*.

* *WGAS was performed using one of these T-cell clones*.

‡ *MiHA specificity of these T-cell clones was determined by recognition of donor EBV-LCL loaded with the MiHA identified using WGAS analysis of another T-cell clone*.

**Table 4:** Overview of identified MiHAs and their characteristics.

**MiHA name**	**SNP**	Peptide polymorphism^*^	**Restriction**	**Number of different TCRBV usages of MiHA T-cell clones**	**T-cells isolated from patient**	**Contribution to immune response^**#**^**
LB-LILRB1-1I	rs1061680	PSPVVNSGGNV[I/T]LQCDSQVA	DQB1*06:02	1	A	60%
LB-RPS4Y	-^‡^	EKTGEHFRLVYDTKGRFAVH	DRB1*03:01	1	B	50%
LB-ABCA5-1R	rs17686569	GKEAIRISGI[R/Q]KTYRKKGEN	DRB1*11:01	1	B	50%
LB-KHNYN-1K	rs3742520	ARGDTYAVEKEGG[K/T]QGGPREMDWG	DRB5*01:01	2	C	9.8%
LB-ZDHHC13-1K	rs2271001	INNRLDLV[K/R]FYISKGAVVDQ	DRB1*15:01	1	C	24%
LB-CTSB-1G	rs1803250	HNFYNVDM[G/S]YLKRLCGTF	DRB1*11:01	2	C	4.9%
LB-LGALS8-1C	rs1041935	DQLDPGTLIVI[C/R]GHVPSDADRF	DRB1*11:01	2	C	7.3%
SLC19A1	rs1051266	DPELRSWR[R/H]LVCYLCFYG	DRB1*15:01	1-2	C	54%
SLC19A1	rs1051266	DPELRSWR[R/H]LVCYLCFYG	DRB1*11:01	1-2	C	
LB-LY75-2R	rs17827158	RAGRPTIKNE[R/K]FLAGLSTDG	DPB1*04:01	6	D	97%
LB-NADK-1K	rs4751	AVHNGLGE[K/N]GSQA	A*03:01	1	D	–

* *Patient type amino acid residues are underlined*.

# *Proportion of alloreactive CD4 T-cell clones recognizing this MiHA as a percentage of all alloreactive CD4 T-cells isolated in that particular patient*.

‡ *Male specific peptide*.

In summary, using WGAS, a total of 9 HLA class II and one HLA class I restricted MiHA were identified as targets for alloreactive T-cells isolated from patients who converted from mixed to full donor chimerism after CD4 DLI.

### Reactivity of MiHA Specific CD4 T-Cells Against Hematopoietic Cells

**Figure 4:**
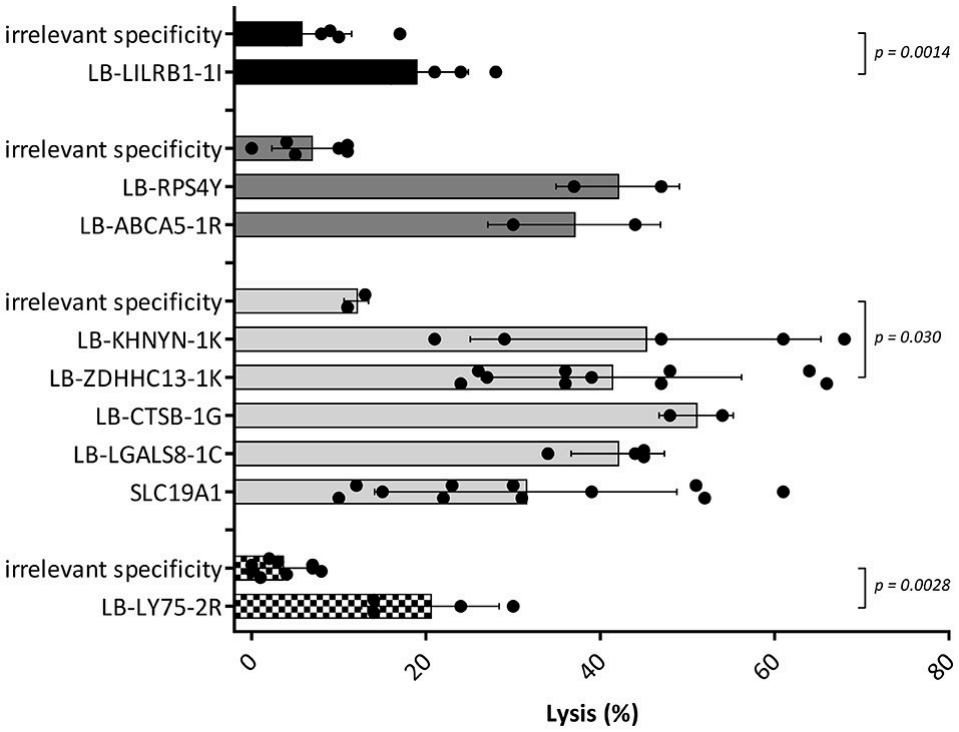
Cytotoxic capacities of MiHA specific CD4 T-cells. Cell lysis calculated after measurement of target cell survival using flowcytometric cell counting after 24 h of incubation with the MiHA specific CD4 T-cells in an effector to target ratio of 10:1. Target cells were patient EBV-LCL and effector cells were MiHA specific CD4 T-cells. CD4 T-cells with irrelevant specificity were used as negative controls. EBV-LCL from patient A, B, C and D are represented by black, dark gray, light gray, and blocked bars, respectively. For all MiHA, individual MiHA specific CD4 T-cell clones were identified that exerted cytotoxic activity against EBV-LCL, resulting in 19–51% specific lysis, although not all differences of the mean lysis by MiHA specific CD4 T-cell clones with background lysis were statistically significant (Mann-Whitney *U*-test) due to low number of experiments.

**Figure 5:**
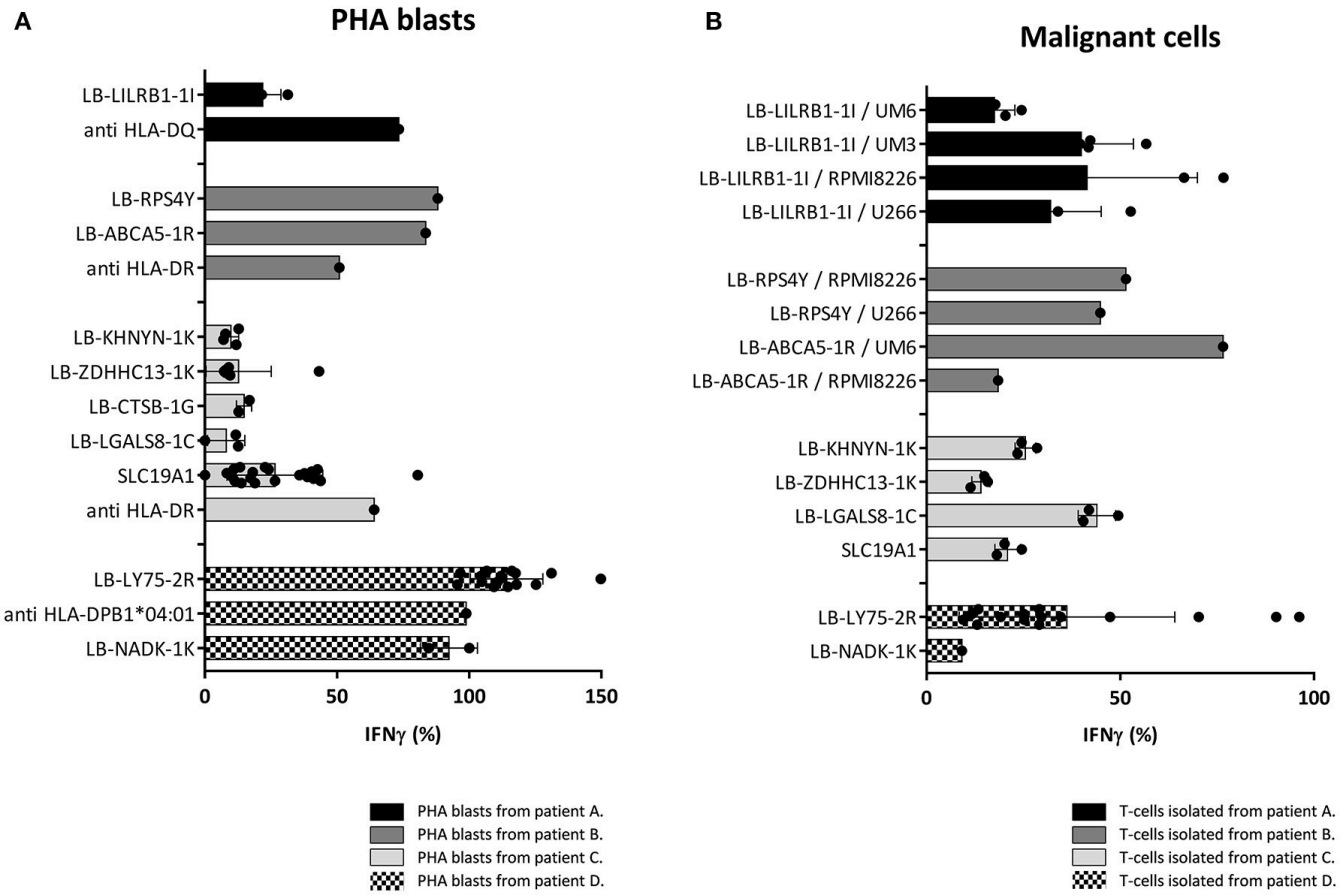
Recognition of PHA blasts and malignant cells by MiHA specific T-cells. Recognition of PHA blasts and malignant cells by MiHA specific CD4 T-cells was measured by IFNγ production, represented as percentage of production after incubation with patient EBV-LCL. Surface expression of HLA class II was confirmed by flow cytometry (data not shown). **(A)** Recognition of patient derived PHA blasts. CD4 T-cells specific for LB-RPS4Y, LB-ABCA5-1R, and LB-LY75-2R as well as CD8 T-cells specific for LB-NADK-1K produced high amounts of IFNγ upon incubation with patient derived PHA blasts. SLC19A1 specific CD4 T-cells produced less IFNγ after incubation with PHA blasts than with EBV-LCL and the other MiHA specific CD4 T-cells did not recognize PHA blasts. T-cell clones directed against the HLA restriction molecule were used as positive controls **(B)** Recognition of MiHA and HLA restriction molecule expressing malignant cells by MiHA specific T-cells. LB-LILRB1-1I specific T-cells were tested against myeloma cell-lines UM-6, UM-3, RPMI8226, and U266. LB-RPS4Y specific T-cells against myeloma cell-lines RPMI8226 and U266 and LB-ACBA5-1R against myeloma cell-lines UM-6 and RPMI8226. T-cells derived from patient C were tested against third party CML cells and T-cells derived from patient D against patient derived AML cells. MiHA specific CD4 T-cells recognizing malignant cells could be detected in all four patients.

Since the induction of MiHA specific CD4 T-cells correlated with conversion from mixed to full donor chimerism as a result of disappearing lymphohematopoietic cells from recipient origin, we investigated whether the isolated MiHA specific CD4 T-cells were capable of exerting cytotoxic activity against recipient hematopoietic cells. MiHA specific CD4 T-cell clones were incubated with patient derived EBV-LCL at an effector to target ratio of 10:1, and after 24 h of co-incubation the numbers of surviving target cells were quantified by flow cytometry. For all MiHA, individual MiHA specific CD4 T-cell clones were identified that exerted cytotoxic activity against EBV-LCL, resulting in 19–51% specific lysis, although not all differences of the mean lysis of all MiHA specific CD4 T-cell clones with background lysis were statistically significant due to low number of experiments (Figure 4). Since conversion of chimerism was most prominent in the T-cell compartment (Table 1), recognition of PHA-stimulated patient T-cells (PHA blasts) by MiHA specific CD4 T-cells was investigated. CD4 T-cells specific for LB-RPS4Y, LB-ABCA5-1R, and LB-LY75-2R as well as CD8 T-cells specific for LB-NADK-1K all recognized PHA blasts as illustrated by high IFNγ release. CD4 T-cells specific for SLC19A1 produced less IFNγ upon incubation with PHA blasts as compared to EBV-LCL, whereas the remaining T-cell clones specific for the other MiHA did not recognize PHA blasts (Figure 5A).

Although all patients were in complete remission of their disease at the time of infusion of CD4 DLI, recognition of malignant cells by MiHA specific CD4 T-cells was investigated to assess their potential to mediate GVL reactivity. Primary malignant cells were only available from patient D and for this reason, third party target cells were used as representative for the malignancies of patient A, B, and C. These target cells expressed the MiHA and HLA restriction element of interest. LB-LILRB1-1I specific CD4 T-cells were incubated with myeloma cell-lines UM-6, UM-3, RPMI8226, and U266 transduced with HLA-DQB1^*^06:02. LB-RPS4Y specific CD4 T-cells were incubated with myeloma cell-lines RPMI8226 [HLA-DRB1^*^03:01 positiv (39)] and U266 transduced with HLA-DRB1^*^03:01. LB-ACBA5-1R specific CD4 T-cells were incubated with myeloma cell-lines UM-6 and RPMI8226 transduced with HLA-DRB1^*^11:01. MiHA specific CD4 T-cells isolated from patient C were incubated with third party bone marrow cells from a patient with CML. Since these third party CML cells were negative for LB-CTSB-1G, recognition of malignant cells by CD4 T-cells specific for this MiHA could not be tested. Reactivity of MiHA specific T-cells from patient D was tested against autologous purified primary AML cells. As shown in Figure 5B, in all four patients, allo-reactive MiHA specific CD4 T-cells were detected that were able to recognize malignant cells.

In conclusion, the results showed that all isolated MiHA specific CD4 T-cells had cytolytic capacity to eliminate recipient hematopoietic cells and that PHA blasts were recognized to a lesser extent than EBV-LCL. Moreover, in all four patients, MiHA specific CD4 T-cells with the potential to recognize malignant cells were found.

### Reactivity of MiHA Specific CD4 T-Cells Against Skin Fibroblasts

**Figure 6:**
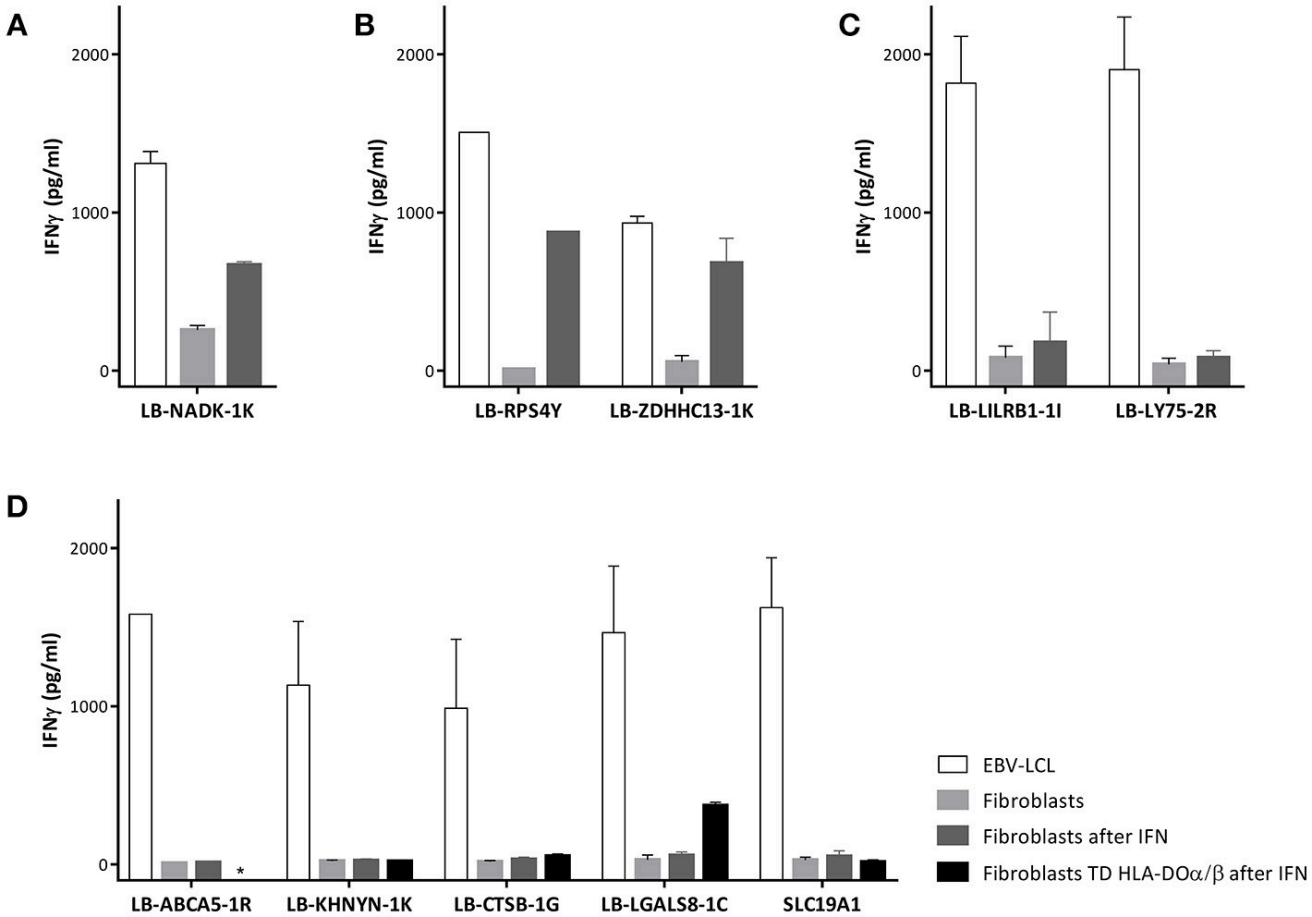
Recognition of fibroblasts by MiHA specific T-cells. T-cell recognition of EBV-LCL, fibroblasts and fibroblasts forced to express HLA class II molecules using interferon gamma (IFN) pretreatment. **(A)** CD8 T-cells specific for LB-NADK-1K recognized fibroblasts already without IFNγ pretreatment. **(B)** CD4 T-cells specific for LB-RPS4Y and LB-ZDHHC13-1K recognized fibroblasts only after IFNγ pretreatment. **(C)** CD4 T-cells specific for LB-LILRB1-1I and LB-LY75-2R failed to recognize fibroblasts due to lack of expression of the MiHA encoding genes. **(D)** CD4 T-cells specific for the remaining 5 MiHA failed to recognize fibroblasts despite detectable gene expression and pretreatment with IFNγ. After transduction with HLA-DOα/β, fibroblasts were recognized by LB-LGALS8-1C specific CD4 T-cells, indicating that presentation of this MiHA was HLA-DO dependent.*, not tested.

To investigate whether non-hematopoietic cells from GVHD target tissues could be targeted under inflammatory conditions due to upregulation of HLA class II expression, we tested recognition of skin derived fibroblasts by the MiHA specific T-cells. Skin fibroblasts were available for two patients (C and D). No fibroblasts were available from patients A and B and we therefore used fibroblasts expressing the MiHA and HLA restriction molecule from third party individuals. Expression of HLA class II molecules on fibroblasts was induced by addition of IFNγ to the culture medium mimicking inflammatory conditions (Supplementary Figure 1). CD4 T-cells directed against allo HLA class II alleles were used as positive controls and all fibroblasts tested were recognized by these clones. Since HLA class I is constitutively expressed on fibroblasts, as expected, CD8 T-cells specific for LB-NADK-1K recognized fibroblasts already without INFγ pretreatment (Figure 6A). CD4 T-cells specific for LB-RPS4Y and LB-ZDHHC13-1K were able to recognize fibroblasts after IFNγ pretreatment (Figure 6B), whereas all other MiHA specific T-cell clones did not recognize (IFNγ pretreated) fibroblasts. CD4 T-cells specific for LB-LILRB1-1I and LB-LY75-2R failed to recognize fibroblasts (Figure 6C) due to the lack of expression of the MiHA encoding genes in fibroblasts (data not shown), while CD4 T-cells specific for the other 5 MiHA did not recognize fibroblasts pretreated with IFNγ (Figure 6D) despite expression of the encoding genes (data not shown). To investigate whether lack of recognition of INFγ pretreated fibroblasts is caused by absence of HLA-DO, fibroblasts were transduced with HLA-DOα/β and tested for T-cell recognition. After transduction and pretreatment with IFNγ, fibroblasts were recognized by CD4 T-cells specific for LB-LGALS8-1C, indicating that presentation of this MiHA is dependent on HLA-DO (32)

These results illustrate that not only restricted expression of HLA class II on hematopoietic cells defines hematopoiesis specific recognition by MiHA specific CD4 T-cell clones, but that even under inflammatory conditions, hematopoiesis restricted recognition is a common phenomenon.

## Discussion

Our results illustrate that purified CD4 DLI, administered with the intention to promote immune reconstitution after TCD alloSCT, can result in conversion from mixed to full donor chimerism without occurrence of GVHD, associated with the development of strong alloreactive CD4 T-cell responses against HLA class II restricted MiHA. Using WGAS we identified 9 MiHA targeted by CD4 T-cells. These MiHA specific CD4 T-cells were likely to be responsible for the hematopoiesis specific immune response. Three months after TCD alloSCT, in the absence of immune suppression, only purified CD4 T-cells were infused, and conversion of chimerism occurred in the first weeks after CD4 DLI in all patients except for patient B in which conversion of chimerism started before, but continued after infusion. During this hematopoiesis specific immune response only alloreactive CD4 T-cells recognizing HLA class II restricted MiHA could be identified in PBMC from 3 of 4 patients. These CD4 T-cells were able to exert direct cytoxicity against hematopoietic target cells in the absence of CD8 T-cells. Reactivity against patient derived activated T-cells (PHA blasts) could be demonstrated by MiHA specific CD4 T-cells in all four patients, strongly suggesting that conversion to full donor chimerism in the T-cell compartment was caused by the alloreactive CD4 T-cells. Although T-cells do not constitutively express HLA class II molecules, they can become targets for CD4 T-cells due to upregulation of HLA class II upon activation (40). From all four patients, MiHA specific CD4 T-cells could be isolated recognizing malignant cells *in vitro*, illustrating the capacity of these T-cells to mediate graft-vs.-malignancy reactivity.

Immune responses by MiHA specific CD4 T-cells are likely to preferentially target cells of hematopoietic origin, since under non-inflammatory conditions HLA class II is predominantly expressed on hematopoietic cells. Under inflammatory conditions, however, non-hematopoietic tissues can become targets for CD4 T-cells due to upregulation of HLA class II expression (31, 40). LB-LILRB1-1I and LB-LY75-2R are MiHA encoded by genes that are not expressed in fibroblasts, and therefore, even under inflammatory circumstances fibroblasts will not be targeted by CD4 T-cells specific for these MiHA. Although all other identified HLA class II restricted MiHA were encoded by broadly expressed genes, only two MiHA specific CD4 T-cell clones were reactive with HLA class II expressing fibroblasts. Apparently, not only expression of the HLA restriction molecule and MiHA encoding gene in GVHD target tissue determine whether these non-hematopoietic cells are targeted by specific CD4 T-cell. We previously showed that, due to the lack of HLA-DO in non-hematopoietic cells, there is a difference in physiology of peptide presentation in HLA class II in hematopoietic cells compared to GVHD target cells (32, 41). Some peptides can only be presented in HLA class II in the presence of HLA-DO, which is expressed in hematopoietic cell types, but not in the majority of non-hematopoietic cells. LB-LGALS8-1C is an example of an HLA-DO dependent antigen since fibroblasts were only recognized by specific CD4 T-cells after enforced expression of HLA-DO. All other MiHA specific CD4 T-cell clones recognized EBV-LCL, but not HLA class II expressing fibroblasts, which may be due to differential avidity of the T-cell clones for these tissues.

Recently, we showed that GVL reactivity without GVHD by MiHA specific CD8 T-cells is not solely determined by tissue distribution of the recognized MiHA, but predominantly depends on magnitude and diversity of alloreactive T-cell responses (33). In the four patients analyzed here, conversion to full donor chimerism after CD4 DLI occurred without GVHD. In patients A and B, T-cell responses with low magnitude and diversity were induced and the majority of T-cells in patient A were specific for an hematopoiesis restricted MiHA. In patient C, magnitude and diversity were higher, but absence of HLA-DO or low avidity of the T-cells for fibroblasts explained lack of GVHD. In patient D there was a high magnitude of a T-cell response, which was directed against an hematopoiesis restricted MiHA. Thus, none of the patients experienced an immune response with both high magnitude and diversity against MiHA that are broadly expressed, resulting in a low risk of GVHD even after upregulation of HLA class II expression.

Patient D developed mild skin GVHD 3 months after CD4 DLI. At this time point, CD8 T-cells specific for LB-NADK-1K were isolated that recognized fibroblasts, while at the earlier time point of conversion of chimerism, more than 99% of alloreactive T-cells isolated were CD4 T-cells that lacked reactivity against fibroblasts. These data suggest that LB-NADK-1K specific CD8 T-cells were responsible for development of this mild GVHD, while LB-LY75-2R specific CD4 T-cells may have mediated conversion of chimerism.

In conclusion, purified CD4 DLI administered 3 months after TCD alloSCT from an HLA-identical sibling donor can lead to conversion of mixed to full donor chimerism due to graft-vs.-patient hematopoiesis reactivity without GVHD by mediating a MiHA specific HLA class II restricted immune response against patient hematopoietic cells, which can also target the malignant hematopoietic counterpart. Even under inflammatory conditions GVL may be separated from GVHD due to the higher susceptibility of hematopoietic cells compared to non-hematopoietic cells from GVHD tissues to be targeted by MiHA specific CD4 T-cells and the limited diversity and magnitude of the induced immune responses.

## Ethics Statement

This study was carried out in accordance with the recommendations of the LUMC Institutional Review Board and national authorities (being CCMO) with written informed consent from all subjects. All subjects gave written informed consent in accordance with the Declaration of Helsinki. The protocol was approved by the CCMO (Centrale Commissie Mensgebonden Onderzoek).

## Author Contributions

PvB, CvB, CH, J-JZ, MG, IJ, and JF designed the research, analyzed results, and wrote the paper. PvB, CvB, SvL-H, WdK, EvE, and SV performed experiments and analyzed results.

### Conflict of Interest Statement

The authors declare that the research was conducted in the absence of any commercial or financial relationships that could be construed as a potential conflict of interest.
