# Supplementary figures and images for: CD4 Donor Lymphocyte Infusion Can Cause Conversion of Chimerism Without GVHD by Inducing Immune Responses Targeting Minor Histocompatibility Antigens in HLA Class II
Source: Front Immunol. 2018 Dec 18;9:3016. doi: 10.3389/fimmu.2018.03016 (PMC6305328; doi:10.3389/fimmu.2018.03016)

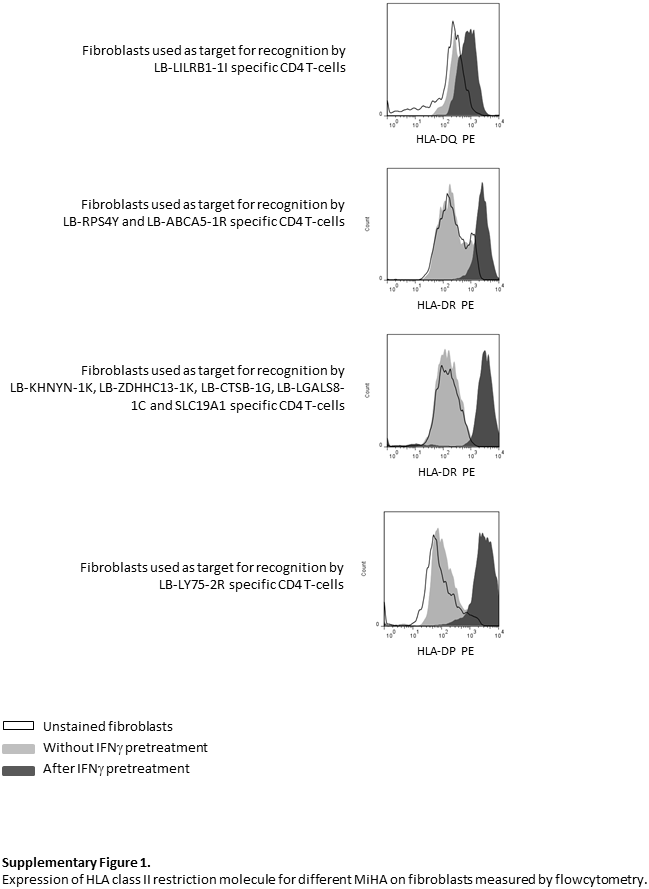

Supplement: Supplementary file 1 [file Image_1.TIF]
